# Advances and new frontiers for immunotherapy in colorectal cancer: Setting the stage for neoadjuvant success?

**DOI:** 10.1016/j.omto.2021.05.001

**Published:** 2021-05-14

**Authors:** Nuttavut Sumransub, Kornpong Vantanasiri, Ajay Prakash, Emil Lou

**Affiliations:** 1 Department of Medicine, University of Minnesota, 420 Delaware St., SE, MMC 480, Minneapolis, MN 55455, USA; 2 Laura and Isaac Perlmutter Cancer Center, NYU Langone Medical Center, 160 E. 34th St., New York, NY 10016, USA; 3 Division of Hematology, Oncology and Transplantation, Department of Medicine, University of Minnesota, 420 Delaware St., SE, MMC 480, Minneapolis, MN 55455, USA

**Keywords:** colorectal cancer, colon cancer, rectal cancer, immunotherapy, cellular therapy, neoadjuvant, solid tumor therapy

## Abstract

Immunotherapy in the metastatic setting has drastically altered the landscape of treatment for various types of malignancy, including colorectal cancer. The category of immune checkpoint inhibitors has especially emerged as a class of therapy predicated on a more comprehensive understanding of immune cell-cancer cell regulation and evolution of the tumor microenvironment over time. Strategies including adoptive cellular therapies, tumor vaccines, and antibodies have also demonstrated the ability to enhance antitumor immunity. In this article, we provide a comprehensive review of the current landscape of immunotherapeutic strategies in colorectal cancer and provide insight into how these strategies may evolve in the next decade and be adapted to more localized forms of cancers of the colon and rectum. We provide particular focus on various combination approaches under investigation for reversing cancer-induced immunosuppression, especially in mismatch repair-proficient/microsatellite-stable colorectal tumors. Finally, we summarize current understanding on a recently identified integral factor in local immune regulation, the colonic microbiome. The aim of this article is to identify current challenges and barriers to improvement and to specify opportunities for applying knowledge in the immunotherapy sphere to rational design of clinical trials intended to improve survival and related outcomes in patients treated in the neoadjuvant setting.

## Graphical abstract

**Figure:**
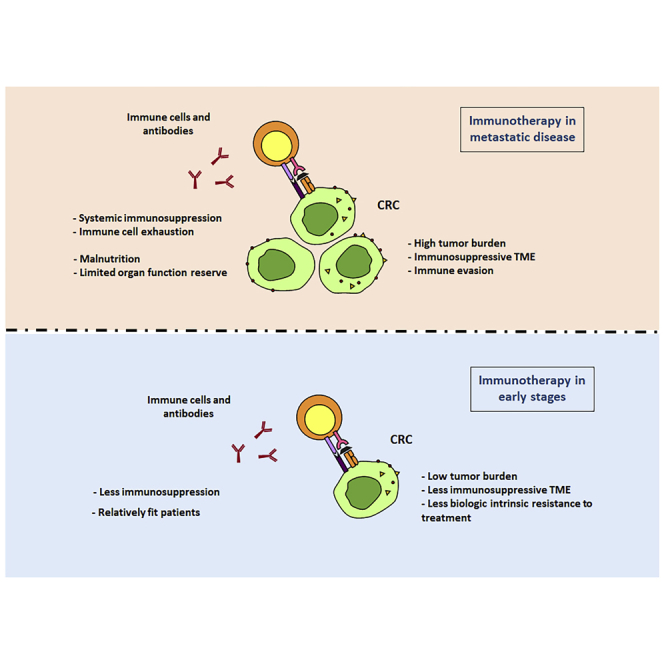

## Teaser

Evolving insight in tumor immunology and the colonic microbiome will allow expansion of current immunotherapeutic strategies to early stages or neoadjuvant settings and to overcome intrinsic biologic resistance, which can improve survival and related outcomes in patients with colorectal cancer.

## Introduction

**Figure 1:**
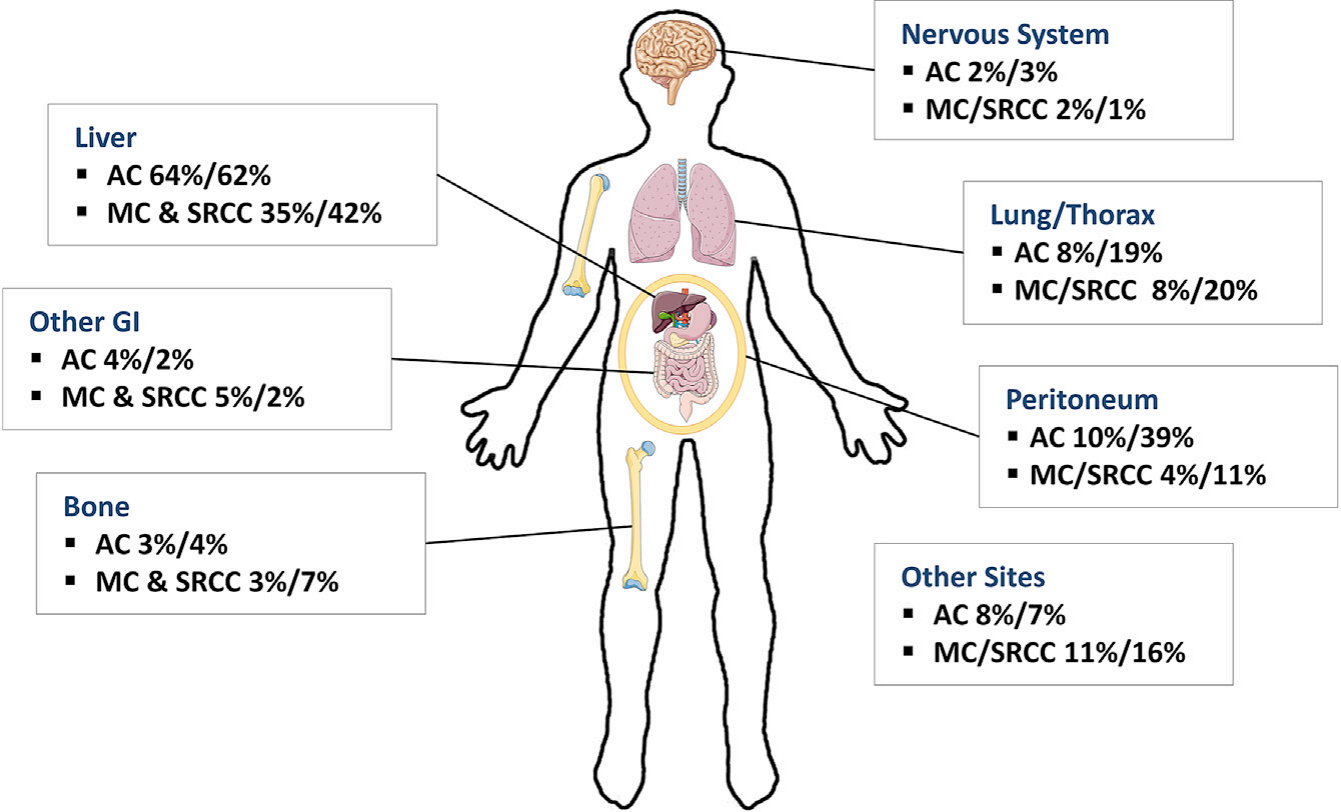
Metastatic pattern of CRC by histology subtypes Percentage represents colon cancer and rectal cancer, respectively. AC, adenocarcinoma; MC, mucinous carcinoma; SRCC, signet ring cell carcinoma. Data adapted from Riihimäki et al.^3^

Colorectal cancer (CRC) is the third most commonly diagnosed cancer and the second most leading cause of cancer death worldwide.^1^ It undoubtedly carries high morbidity, mortality, and a significant burden for the healthcare system.^2^ Although standard treatment regimens, including surgery, radiotherapy (RT), and chemotherapy, are effective for locoregional diseases, the treatment of metastatic diseases remains challenging (Figure 1). Cytotoxic forms of chemotherapy have provided the mainstay backbone of treatment strategies for patients with advanced CRC for the past several decades, providing incremental improvements in overall survival (OS) rates through combination therapy with or without addition of biological targeted agents. However, the 5-year survival rate of metastatic CRC (mCRC) is still relatively low at 14%, with a mean OS of approximately 30 months compared to localized and regionalized CRCs.^4^^,^^5^

Many novel therapeutic measures have been considered and investigated in recent years to bridge this gap. Implementation of immunotherapy has long been a desired goal in the field of cancer research due to its promise for tailoring treatments to individual patients while also promising potential for inducing a longer-term form of immune surveillance that theoretically could decrease risks of future recurrence of disease. The reality of biologic discovery and implementation of immunotherapy has been a longer process than anticipated but has certainly entered more mainstream acceptance over the past decade with randomized controlled trials (RCTs) demonstrating success in the form of longer survival for patients with metastatic melanoma and non-small cell lung cancer, most prominently. However, the extent of that success has not immediately transferred as well to many other forms of systemic malignancies, including cancers of the gastrointestinal tract, and CRC quite specifically. Thus far, evidence supporting the use of immune checkpoint inhibitors (ICIs) is most abundant in cases of metastatic treatment-refractory cases of gastric cancer and hepatocellular carcinoma;^6^^,^^7^ what has made those approvals interesting is that the response rate has been independent of biologic marker status, such as microsatellite instability-high (MSI-H) or programmed cell death-ligand 1 (PD-L1) expression.

**Figure 2:**
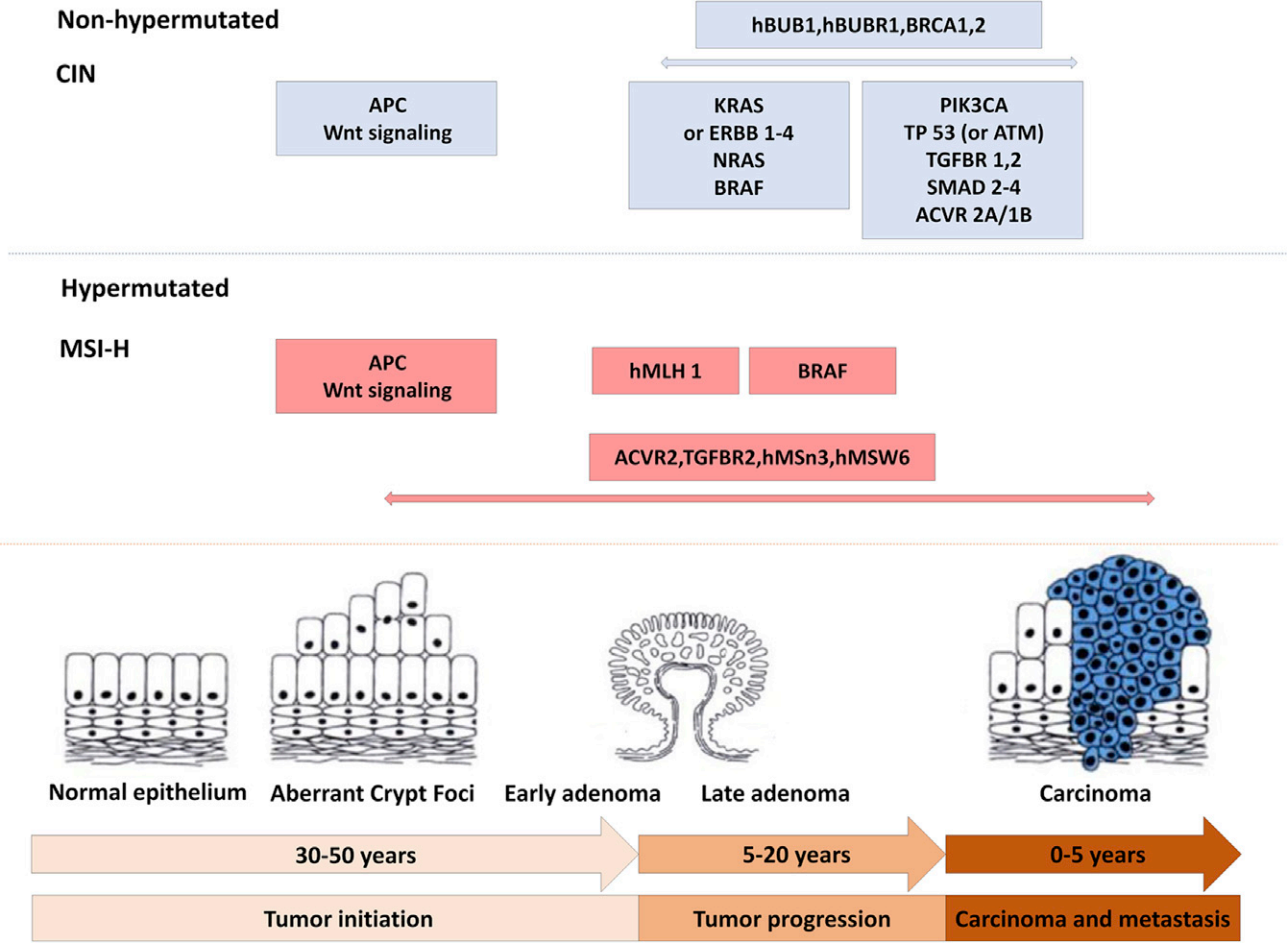
Adenoma-carcinoma sequence with genetic alteration pathways Chromosomal instability pathway (CIN) characterized by somatic copy number alterations with aneuploidy tumors. MSI-H is hypermutated and characterized by frameshift mutation of target genes.

Development of CRC involves complex processes accumulating genetic mutations, resulting in the heterogeneity of response to treatment (Figure 2). The use and approval for ICIs in CRC are thus far predicated on expression deficient mismatch repair (dMMR) with MSI-H based on landmark trials published in the past decade; however, of all patients with CRC, only 5%–15% of this population have tumors with this predictive biomarker, leaving the vast majority of patients with CRC without opportunity for therapeutic efficacy with immunotherapy at the current time.^8^ If more predictive biomarkers of immunotherapy success can be identified, that finding would significantly expand the number of patients with CRC who could benefit beyond the current paradigm.

Even further, as investigations in this field to date have focused on patients with metastatic drug-refractory forms of CRC, findings thus far demonstrating exceptional response rates in some subsets of tumors and patients point to a fertile opportunity to also reexamine regimens currently being used for the neoadjuvant setting for resectable and locally advanced forms of rectal carcinomas, as these patients reside within a window of opportunity to succeed in curative intent treatment due to a relatively limited extent of measurable disease. Furthermore, as a majority of cases of recurrent disease will manifest with distant metastatic disease, consideration of upfront immunotherapy offers an additional theoretical benefit by adding an immune-surveillance component that would further reduce recurrence risk in the long term and also potentially lowering risk of short- and long-term adverse side effects compared to cytotoxic chemotherapies in certain patient populations. To get to that point, there are major challenges and unanswered questions that will need to be addressed in well-designed human trials. The many variables to consider include and are not limited to variability of the host immune system between individual patients and patient populations, co-morbid conditions, and varying immunological milieu within tumor microenvironments (TMEs) that will result in variation of responses and require further investigation. In recent years, a spotlight has been placed on the role of the gut microbiome on an individual host’s response to immunotherapy, with ongoing research underscoring this previously unknown wild card.^9^^,^^10^ Accurate identification of all of these key factors for improving therapeutic efficacy of immunotherapy will be paramount. In this review, we summarize the update on immunotherapy in treating mCRCs, highlight the relevant clinical trials that elucidate the efficacy and safety of these currently emerging immunotherapies, and cover the influence of gut microbiome on the efficacy of immunotherapy in CRC treatment.

### Current treatment for mCRC

Understanding the current context of treatment of patients with mCRC, which has been in place for nearly two decades, helps to provide a foundation of understanding for how translation can occur to the neoadjuvant setting. There are multiple combination regimens available as part of current standards of care for mCRC. These generally include a 5-fluorouracil (5-FU) backbone-based regimen in combination with either the platinum compound oxaliplatin (FOLFOX) or topoisomerase I inhibitor irinotecan (FOLFIRI) or all three (FOLFOXIRI); either of these chemotherapy doublet or triplet regimens may be combined in some patients with biologic agents targeting vascular endothelial growth factor (VEGF) or epidermal growth factor receptor (EGFR), the latter of which has biologic activity only in tumors harboring wild type but not mutant isoforms of RAS.^11^, ^12^, ^13^, ^14^, ^15^, ^16^, ^17^, ^18^, ^19^, ^20^, ^21^, ^22^, ^23^, ^24^, ^25^, ^26^, ^27^, ^28^, ^29^, ^30^, ^31^, ^32^, ^33^, ^34^, ^35^, ^36^, ^37^, ^38^, ^39^, ^40^, ^41^, ^42^, ^43^, ^44^, ^45^ For over a decade, the use of EGFR inhibitors cetuximab or panitumumab, monoclonal antibodies against EGFR that can be effective as single agents or in combination with 5-FU-based therapies for cases of mCRC, has been a mainstay of what can be considered “passive immunotherapy.” EGFR activation mediates cell proliferation and tumor progression via downstream signaling through the RAS/RAF/mitogen-activated protein kinase (MAPK) pathway.^46^ Blockade of EGFR has been used extensively in the metastatic setting in cases where tumors are proven to be wild type for RAS expression, and although response rates can be higher in some settings with addition of EGFR inhibition, its use in the upfront setting for locally advanced rectal cancers has been shown to cause increased morbidity without clinical benefit.^47^

### Immunotherapy in CRC

**Figure 3:**
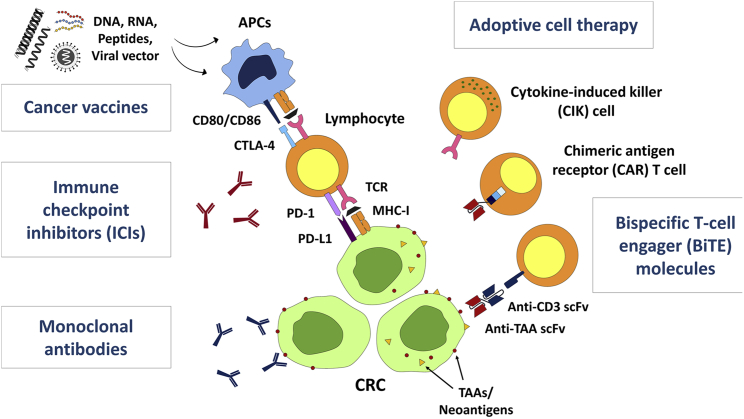
Current state of immunotherapy in CRC Major modalities of immunotherapy in clinical trials include monoclonal antibodies, immune checkpoint inhibitors, cancer vaccines, adoptive cell therapies, and bispecific T cell engagers. The majority of the recent studies are investigating the efficacy of combination strategies and moving the application toward earlier-stage and neoadjuvant settings.

Various strategies in activating immunity against cancer have been explored in CRC (Figure 3). The mechanisms of how the endogenous immune system is altered at the cellular level by tumors circumventing immune surveillance have been better elucidated this century. However, how the complex interplay between cancer cells and TME reduces immune function and promotes growth of the tumor remains an active area of investigation that will be needed to develop next-generation classes of immunotherapeutic agents. Understanding how this will affect tumors such as CRC at the TME as well as molecular levels is crucial, most especially in the context of the immune infiltration aspect of tumor biology that shapes whether or not tumors become “hot” or “cold,” in other words, responsive to immunotherapeutic checkpoint inhibitors.

#### ICIs

The emergence of TME resistance to immune surveillance is mainly induced through several immune checkpoint signaling cell surface receptors, most prominently programmed death-1 (PD-1), PD-L1, and cytotoxic T-lymphocyte-associated antigen-4 (CTLA-4).^48^ These immune checkpoint molecules act as negative regulators of the immune response. CTLA-4 is a central regulator in the early stages (priming phase) of T cell activation in lymphoid organs. When paired with B7 protein on activated antigen-presenting cells (APCs), the CTLA4-B7 interaction inhibits T cell activation and minimizes anti-tumor immune response.^49^ By contrast, PD-1 acts as a peripheral checkpoint and interacts with two family members of B7 protein, PD-L1 and PD-L2, to limit effector T cell activity in the peripheral tissues and TME.^50^ Tumor cells can also upregulate PD-L1, which causes T cell exhaustion and leads to activated T cell apoptosis.^51^ Infiltration of T cells has long been considered a prognostic biomarker;^52^ tumor-infiltrating T cell counts correlate with improved survival rate and lower risk of cancer recurrence.^53^^,^^54^ Within this background, ICIs were developed to enhance function and activation of these T cells; prevent T cell dysfunction and apoptosis; and antagonize immunosuppressive effects mediated by CTLA-4, PD-1, and its ligand PD-L1.^55^

Initial studies of ICIs in CRC demonstrated very limited activity in unselected CRC populations.^56^ A phase I study with the anti-PD1 antibody nivolumab found that one patient with dMMR/MSI-H CRC showed response to treatment at 21 months and achieved a complete response (CR) after re-treatment.^57^^,^^58^ The result led to further investigation and in a phase II trial of pembrolizumab (KEYNOTE-016), another anti-PD1 antibody, demonstrated an objective response rate (ORR) of 40% and immune-related, progression-free survival (PFS) rate at 78% for dMMR CRC with 0% ORR and 11% immune-related PFS rate for pMMR CRC.^59^ A trial of nivolumab in dMMR-MSI-H mCRC (CheckMate 142) later revealed that 31% of patients achieved an investigator-assessed objective response, and 69% had disease control for 12 weeks or longer.^60^ Combinations of nivolumab and ipilimumab were also evaluated in this trial and showed investigator-assessed ORRs of 55%, and disease control rate (DCR) for ≥12 weeks was 80%.^61^ Largely on the basis of the overall compiled clinical trial data, in 2018, pembrolizumab and nivolumab were approved by the US Food and Drug Administration (FDA) for the treatment of patients with dMMR-MSI-H mCRC refractory to 5-FU, oxaliplatin, and irinotecan. Pembrolizumab was later approved for first-line treatment of patients with unresectable dMMR-MSI-H mCRC in June 2020 based on the KEYNOTE-177 study that showed significantly longer PFS compared to the chemotherapy group (median, 16.5 versus 8.2 months), with fewer treatment-related adverse events (AEs).^62^ Notably, out of interest in potential applicability for future studies using pembrolizumab in neoadjuvant settings, the overall response rate (combined partial response [PR] and CR) was 43.8%, as opposed to 33.1% in the chemotherapy group.

There are also trials investigating the effects of the addition of atezolizumab to FOLFOX plus bevacizumab (ClinicalTrials.gov: NCT02997228) or to FOLFOXIRI plus bevacizumab (ClinicalTrials.gov: NCT03721653) in mCRC. In the second-line treatment setting, pembrolizumab showed a 33% ORR; a trial comparing the effect of anti PD-L1 antibody, avelumab, to standard chemotherapy is underway (ClinicalTrials.gov: NCT03186326).^63^ Common ICI-related AEs include fatigue, diarrhea, pruritus, thyroid dysfunction, hepatitis, arthralgia, fever, and rash. Patients who received a combination of ICIs have observed an increase in grades 3−4 AEs. This constellation of symptoms, including its wide range of possible grade and extent of the AEs, is cause for strong discussion prior to design of clinical trials utilizing ICI in the neoadjuvant settings. As compared to use of upfront cytotoxic chemotherapy regimens, such as FOLFOX, the relatively short and well-defined prescribed duration of ICI over several months prior to, for example, total mesorectal excision of locally advanced rectal carcinomas may allow for maximal benefit while minimizing these potential toxicities.

Studies in early-stage melanoma showed that neoadjuvant ipilimumab + nivolumab expands more tumor-resident T cell clones than adjuvant application with a pathological response; this finding prompts consideration of approach and implications in the setting of locally advanced CRC as well.^64^^,^^65^ Possible benefit is based on the rationale that the dMMR-MSI-H phenotype is more common in earlier-stage cancers than in mCRC. Although limited in numbers, some of the growing body of data giving rationale for use of ICI in the neoadjuvant setting for CRC comes from case series reporting favorable pathologic responses in neoadjuvant settings in MSI-H gastrointestinal tumors.^66^^,^^67^ One recently published study showed 100% (95% confidence interval [CI]: 86%–100%) pathological response in dMMR and 27% (95% CI: 8%–55%) in pMMR in early-stage CRC when using neoadjuvant ipilimumab and nivolumab.^68^ Thirteen percent of patients experienced grades 3−4 treatment-related toxicity, emphasizing the importance of risk-benefit evaluation for the use in this setting. A phase III study is currently investigating the result of combining atezolizumab with FOLFOX in stage III CRC (ClinicalTrials.gov: NCT02912559; ATOMIC trial) with another phase II study planned to study the efficacy of pembrolizumab in early-stage colon cancer (ClinicalTrials.gov: NCT04231562). The combination of ICIs to neoadjuvant therapy in CRC is also under investigation based on the hypothesis that chemoradiotherapy may increase the neoantigen formation and increase the efficacy of ICIs (ClinicalTrials.gov: NCT04293419, NCT04017455, NCT03127007, NCT02921256, NCT01409755, NCT04083365, and NCT04621370).

#### Tumor vaccination

Therapeutic cancer vaccines have been applied in various cancer types to enhance immune response and immunological memory to prevent recurrence.^69^ The general mechanism includes activation of immune cells after exposure to specific target antigens known as tumor-associated antigens (TAAs) that are overexpressed in tumors. Recently, there is an increasing interest in antigens arising from spontaneous mutations in cancer cells, known as tumor-specific antigens (TSAs) or neoantigens, for their potential ability to predict susceptibility to immunotherapy products. The process for identifying neoantigens is specific to individual tumors and is time and resource intensive. Despite the need for a high-throughput sequencing method and bioinformatics algorithms to predict peptide binding to major histocompatibility complex (MHC) molecules, these antigens offer personalized approaches to cancer treatment and are under investigation in multiple clinical trials (ClinicalTrials.gov: NCT03552718, NCT03639714, NCT03953235, and NCT04147078). Various methods of antigen delivery have been investigated including molecular-based vaccines (peptide, DNA, and RNA), cell-based vaccines (tumor cells and dendritic cells [DCs]), and vector-based vaccines (viral, bacteria, and yeast). These have shown mixed results with limited efficacy in improving clinical outcomes.^70^ Toll-like receptor (TLR) agonists, especially TLR7/TLR8, are currently under investigation for their immunostimulatory properties and could offer a combination strategy with cancer vaccine as an adjuvant-like signal.^71^

#### Adoptive cell therapy (ACT)

ACT is a novel therapeutic strategy that has made great strides over the past several decades. The strategy allows selection, activation, and expansion of effector immune cells (EIC) *in vivo* following infusion into patients. Currently, there are two main classes of ACT in the process of development for clinical use, as follows.

##### Tumor-infiltrating lymphocytes (TILs) and cytokine-induced killer (CIK) cells

TILs are lymphocytes that infiltrate within tumors or at the tumor margin. The presence of TILs is associated with better prognosis due to selective antitumor activity.^72^ Whereas TILs show efficacy in known immunogenic cancers such as melanoma and renal cell carcinoma, results in application in CRC have been less certain.^73^ Some of the challenges of TILs inherent to CRC tumors are the limited number of cells in CRC tumors and relatively suppressed immune cell function.^74^ The development of CIK cells in combination with TAA-bearing DCs has overcome these hurdles and has been shown to increase PFS and OS.^75^ Ongoing studies in ACT now focus on a combination strategy with drugs or ICIs to enhance efficacy (ClinicalTrials.gov: NCT01420874, NCT02487992, NCT03904537, and NCT04282044). The strategy that combines ACT with conventional chemotherapy treatment has also shown promising results (ClinicalTrials.gov: NCT03950154).^76^

Advances in deriving TILs from solid tumors, including from CRC, in combination with improvements in gene-editing techniques including the CRISPR-Cas9 system, have collectively opened the door toward enhancing potential for this therapeutic strategy in treatment of patients with CRC. Novel checkpoint targets have emerged beyond PD-L1 for other gastrointestinal cancers (e.g., gastroesophageal) and MSI for CRC and others. Evolution and improvement of gene-editing techniques that have higher efficiency as well as decreased off-target editing rates, in combination with such novel immune checkpoints, represent a new frontier for therapeutics that, if successful in the metastatic setting, may provide a foundation for use in the neoadjuvant setting in the not-too-distant future. One such example is already in clinical trial at our institution and comprises CRISPR-Cas9 inhibition of the intracellular immune checkpoint CISH. Inhibition of CISH has been achieved at >90% success rates, and the preclinical research at the University of Minnesota in collaboration with researchers at the National Cancer Institute led to a current clinical trial, opened in May 2020, enrolling patients with metastatic gastrointestinal cancers refractory to at least one line of treatment (Clinicaltrials.gov: NCT04426669). This combined phase I/II trial will determine both safety as well as efficacy of this new approach to ACT; if successful, it is conceivable that this approach in the neoadjuvant setting could be utilized to induce significant tumor regression for an overall intent-to-cure approach combined with surgical resection and possibly lead to higher rates of clinical CR using current modalities. Furthermore, the concept of immune surveillance using this ACT method has intriguing opportunities for also inducing a longer-term form of specific anti-tumor immunity in any given individual patient, introducing an interesting possibility of simultaneously decreasing the risk of short- and long-term recurrence beyond the current capabilities of cytotoxic chemotherapy given in the adjuvant setting.

##### Chimeric antigen receptor (CAR) cell therapy

CARs are bioengineered receptors recognizing specific antigens by a single-chain fragment variant, followed by downstream triggering of an intracellular signaling pathway leading to effector function and immune response. CAR-T cell therapy strategies are gaining enormous interest due to their success in treatment of hematologic malignancies; thus applications in the treatment of solid tumors are being heavily investigated, and such trials are being watched with great interest. Beside the efficacy in cancer cell killing, the method also has a major advantage for its MHC-independent action that can overcome immunological escape by MHC class I downregulation by tumor cells. Whether or not this modality will prove efficacious in CRC of any stage remains unknown.

Carcinoembryonic antigen (CEA) is an attractive target for immunotherapy in CRC as the molecule is frequently overexpressed in over one-half of patients with CRC. One study recruited three patients and demonstrated a significant decrease in serum CEA level, with one patient having an objective response in metastatic lesions following T cell treatment.^77^ However, a severe transient inflammatory colitis that represented a dose-limiting toxicity was induced in all three patients. A study by Zhang et al.^78^ demonstrated that most patients who experienced progressive disease (PD) in previous treatments have stable disease after CAR-T therapy, with two out of ten patients showing tumor shrinkage. Currently, multiple studies in CEA CAR-T cells are in clinical trials (ClinicalTrials.gov: NCT02850536, NCT03682744, NCT04348643, and NCT04513431).

Natural killer group 2D ligands (NKG2DLs) are gaining interest, as they are commonly overexpressed in cancer and can be a target for NKG2D receptor-based CAR-T cells^79^ (ClinicalTrials.gov: NCT03310008, NCT03370198, NCT03692429, NCT04107142, and NCT04550663). Other potential targets undergoing clinical studies include EGFR (HER-2) (ClinicalTrials.gov: NCT03542799, NCT03740256, and NCT04660929), mesothelin (ClinicalTrials.gov: NCT04503980), guanylyl cyclase C (GUCY2C), and mucin 1 (MUC1), with most of the studies still in an animal model or early phase of clinical trials.^80^ CAR-T cell-targeting neoantigen can overcome off-target actions and minimize side effects, as the strategy can target antigens that are exclusively expressed on the surface of that patient’s tumor cells (ClinicalTrials.gov: NCT03970382). Major challenges in the application of CAR cells in solid tumors include physical barriers surrounding cancer cells leading to insufficient infiltration, immunosuppressive TME, and barriers on logistics and cost of the treatment.

#### Bispecific T cell engagers (BiTEs)

BiTEs are artificially produced antibodies containing two distinct single-chain variable regions. The mechanism is through binding of the target antigen by one antibody fragment and engaging the T cell by another fragment specific to CD3. This action increases T cell infiltration and generates a highly inflamed TME leading to cancer cell apoptosis.^81^ CEA is a TAA commonly found to be overexpressed in CRC cells and is a target for preclinical and clinical studies for BiTE both as monotherapy (ClinicalTrials.gov: NCT02324257) or combination therapy (ClinicalTrials.gov: NCT02650713 and NCT03866239).^82^

#### Oncolytic viruses

Virotherapy for cancer treatment is another evolving field that harnesses unique biologic characteristics of the viruses. Oncolytic viruses are designed to preferentially infect and replicate in the cancer cells, causing cell lysis, and induce host anti-tumor immune responses.^83^ Furthermore, immunostimulation and tumor suppression can be achieved using genetic engineering technology to arm viruses with certain molecules. The therapy has shown efficacy in various types of solid tumors, and talimogene laherparepvec, genetically modified herpes simplex viruses expressing granulocyte-macrophage colony stimulating factor (GM-CSF), was approved by the FDA in October 2015 for the treatment of melanoma.^84^ However, despite demonstrations of safety in early-phase clinical trials, only limited evidence on the efficacy of virotherapy is available, and there is currently no approved oncolytic virus therapy for mCRC.^85^

Methods have been employed to increase efficacy and safety for this therapy option. Combination of pelareorep (oncolytic reovirus) with FOLFOX/bevacizumab was tolerable with an increased ORR, but PFS was inferior.^86^ Oncolytic reovirus in combination with FOLFIRI and bevacizumab (ClinicalTrials.gov: NCT01274624) and recombinant vaccinia virus in combination with irinotecan (ClinicalTrials.gov: NCT01394939) are also under evaluation. Pre-clinical studies have also provided promising results for oncolytic virus combination with ICIs and were recently translated into clinical trials in CRC^87^^,^^88^ (ClinicalTrials.gov: NCT03206073 and NCT04348916). A combination therapy of binary oncolytic adenovirus in combination with HER2-specific autologous CAR-T cells is currently under investigation as well (ClinicalTrials.gov: NCT03740256).

### ICIs in proficient MMR (pMMR): microsatellite-stable (MSS) mCRC

Effectiveness of ICI treatment is limited to the dMMR-MSI-H subset, which represents around 15% of localized CRC and about 4% of mCRC.^89^ In contrast, there was no objective response observed in the pMMR/MSI-low (MSI-L) group.^59^ A more recent study on ICI treatment for pMMR mCRC by Chen et al.^90^ showed significantly prolonged OS (6.6 months versus 4.1 months) and better DCR (22.7% versus 6.6%) in patients with advanced refractory CRC who received durvalumab plus tremelimumab compared to best supportive care.

**Figure 4:**
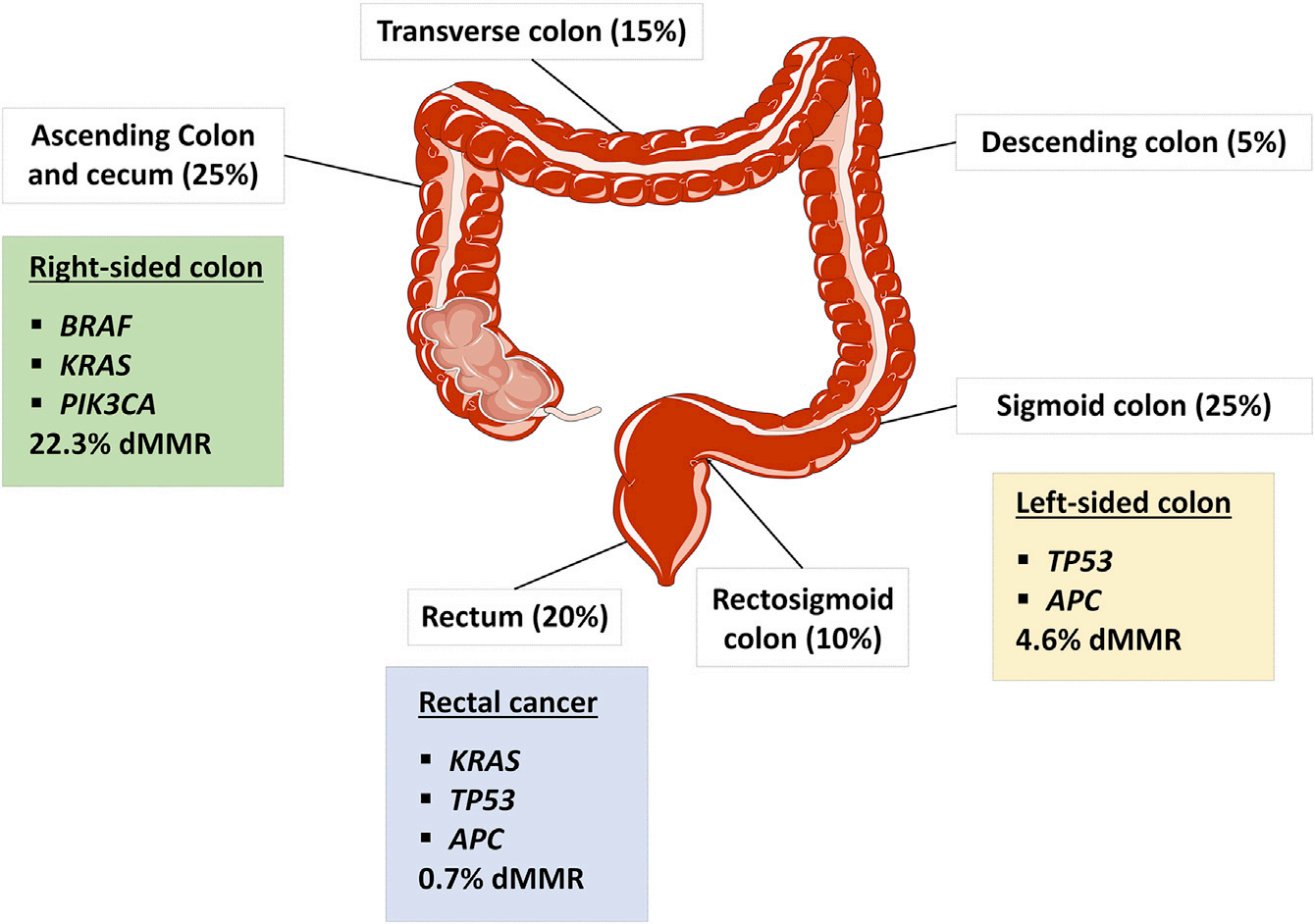
Anatomic distribution of CRC and their pattern of genetic mutation Genetic mutations are different in frequencies based on the location, and primary R-sided colon cancer has the highest rate of dMMR.

The differences in TME composition and immune cell distributions between dMMR-MSI-H and pMMR-MSI-L tumors contribute to response rate to therapy and prognosis.^91^ Interestingly, the distinct anatomic locations of tumors exhibit different molecular characteristics and clinical behaviors^92^ (Figure 4). Deficiency of MMR systems leads to high mutational burden, and expressions of mutation-generated neoantigens recruit immune cells. It has been proven that dMMR tumors are enriched with T helper 1 (Th1) cells and cytotoxic T lymphocytes, and thus, this landscape may explain the superior biological response to ICIs.^93^ Combination strategies have been investigated to reverse the resistance caused by poor antigenicity of pMMR mCRC with the main principle in increasing antigen exposure to immune cells.^94^^,^^95^ The results to date have shown only modest efficacy or mixed results; thus, novel strategies are still in need of development to apply in clinical settings for pMMR-MSI-L tumors.

Immunogenic cell death induced by chemotherapy leads to antigen release and immune activation, which could sensitize tumor cells to ICI therapy.^96^ Multiple studies combine chemotherapy with anti-VEGF, as its inhibition can lead to reversal of myeloid-derived suppressor cell (MDSC), tumor-associated macrophage (TAM), and regulatory T cell (Treg) functions and enhance T cell infiltration and interaction with APCs.^97^^,^^98^ However, results from the MODUL trial (ClinicalTrials.gov: NCT02291289) and BACCI trial (ClinicalTrials.gov: NCT02873195) revealed minimal effects on PFS and OS.

RT has long been postulated to activate antitumor immunity at distant sites through what has been termed “the abscopal effect.” A study in melanoma models also showed that this combination can reverse treatment resistance due to upregulation of PD-L1 on cancer cells.^99^ A study evaluating the efficacy of pembrolizumab plus RT in pMMR mCRC reported an interim ORR at 9% (1 of 11) in the RT cohort with no responses observed in the tumor ablation cohort.^100^ Another RCT combining ipilimumab and nivolumab with RT in MSS mCRC reported a DCR of 17.5% (7/40) with a 7.5% (3/40) ORR.^101^

Monoclonal antibodies targeting EGFR (cetuximab or panitumumab) are a standard of care for treatment of wild-type RAS mCRC. A previous *in vitro* study revealed a subgroup of patients who carry certain Fc gamma receptor (FcγR) polymorphisms with higher antibody-dependent cell cytotoxicity (ADCC) activity has significantly longer PFS.^102^ This indicates significant effects of ADCC and NK cell activation, which could be potentiated by ICIs. A phase Ib/II study combining cetuximab with pembrolizumab reported tolerable side-effect profiles with 6/9 patients achieving stable disease lasting ≥16 weeks.^103^ Results from the phase II AVETUX trial, which has a majority of patients with MSS mCRC enrolled, revealed ORR at 79.5%, including 6 CRs and 25 PRs.^104^

Regorafenib, a multi-kinase inhibitor, possesses pleiotropic effects in inhibiting angiogenesis and immunomodulation, mainly through TAM inhibition.^105^ The REGONIVO trial, a phase Ib trial of regorafenib plus nivolumab for gastric and CRC, determined that regorafenib 80 mg plus nivolumab had a manageable safety profile.^106^ REGOMUNE, a phase II trial investigating the combination of regorafenib plus avelumab, reported stable disease for 23 patients (53.5%) and PD for 17 patients (39.5%) with median PFS and OS at 3.6 months (95% CI: 1.8−5.4) and 10.8 months (95% CI: 5.9−not applicable [N/A]), respectively. High baseline infiltration by TAMs was significantly associated with adverse PFS, whereas increased tumor infiltration by CD8^+^ T cells from baseline was significantly associated with a better outcome.^107^

Inhibition of mitogen/extracellular signal regulated kinase (MEK), a downstream effector of the RAS-MAPK pathway, was found to result in upregulation of PD-L1 and have synergistic effects with the PD-1 inhibitor in preclinical models.^108^^,^^109^ However, the result of the IMBLAZE370 trial studying the effect of cobimetinib did not meet its primary endpoint of improved OS with atezolizumab plus cobimetinib or atezolizumab versus regorafenib.^110^

Currently, there are extensive ongoing trials exploring knowledge in the field to improve the efficacy of immunotherapy.^111^^,^^112^ Examples of other ICI combination strategies include tyrosine kinase inhibitors (TKIs), indoleamine-2,3-dioxygenase-1 (IDO1) inhibitor, CCR5 inhibitors, cytokines and TLR ligands, oncolytic virus, and ACT.

### Immunotherapy and the colon microbiome

The microbiome has been well described in CRC carcinogenesis^113^, ^114^, ^115^, ^116^ and is recognized as increasingly important in colon immune regulation.^117^ Several studies have implicated immune modulation as the mechanism of this effect,^118^, ^119^, ^120^, ^121^, ^122^ with an abundance of the species *Fusobacterium nucleatum* correlated with CD3^+^ TIL depletion^119^^,^^121^^,^^122^ and macrophage suppression.^123^ Given the importance of tissue-resident T cells and macrophage activation in immunotherapy treatment and response and historical data demonstrating the importance of the microbiome in chemotherapy response,^124^^,^^125^ these data drove interest in evaluating the relationship between the microbiome and cancer immunotherapy.

Given the limitations in CRC-directed immunotherapy, early studies focused on the interaction between the microbiome and anti-PD-1/CTLA-4 therapies in metastatic melanoma.^126^^,^^127^ In a mouse model, tumor growth suppression and anti-PD-L1 antibody activity were found to be associated with *Bifidobacterium* abundance.^126^ Co-housing, fecal transfer, and *Bifidobacterium*-containing pro-biotics were all found to be able to transfer this effect. Evaluation of the immune environment revealed that enhanced tumor-specific CD8^+^ T cell activity and DC maturation in correlation with *Bifidobacterium* abundance were the keys to this response, reflecting earlier findings on the microbiome and carcinogenesis.

Similarly, anti-CTLA-4 antibody efficacy against mouse-model sarcoma and melanoma was found to be promoted by the presence of specific *Bacteroides* taxa and abrogated by antibiotic induced dysbiosis.^128^ In particular, transfer of the taxa *Bacteroides thetaiotaomicron*, *Bacteroides fragilis*, and *Burkholderia capacia* was able to restore anti-tumor activity of anti-CTLA-4 antibodies in germ-free mice. Further, these taxa were noted to induce Th1 immune response and promote DC maturation. This particular study went further, showing that human-mouse fecal transfer of feces enriched for *B. fragilis* and *B. thetaiotaomicron* induced a marked response to anti-CTLA-4 therapy.

Subsequent human studies identified additional taxa involved in immunotherapy treatment response, including *Ruminocaccaceae faecalibacterium*, and *Akkermansia muciniphila*.^129^, ^130^, ^131^ In patients with metastatic melanoma, treatment response to anti-PD-1 therapy was associated with a broader overall diversity of commensal bacteria and an increased relative abundance of *Faecalibacterium*,^130^ and in patients treated with anti-CTLA-4 antibodies, enrichment of *Faecalibacterium* was associated with significantly longer PFS/OS.^129^ Similarly, abundance of *A. muciniphila* was positively associated with a treatment response in patients with multiple advanced cancers.^131^

Given these promising results, more recent studies have focused on identifying a subset of bacteria that may modify the tumor immune environment^132^ and promote an immunotherapy response^133^ (Table 1). Cremonesi and colleagues^132^ showed that a subset of low-abundance microbial taxa was associated with increased CD3^+^ lymphocyte infiltration in clinical CRC samples. Specifically, the abundance of the families *Lachnospiraceae* and *Ruminococcaceae* was correlated with expression of CCR5 and CXCR3 chemokines, the latter of which is associated with tissue T cell trafficking.^134^ These data, along with the prior mouse studies on *Ruminococcaceae* family members, suggest that microbiome-based therapies may be useful in priming tumors for response to immunotherapies. Another landmark study demonstrated that a group of 11 low-abundance bacteria was necessary and sufficient to enhance anti-PD-1 antibody activity against a mouse xenograft colon cancer model.^133^ This effect appears to be modulated through increased tumor infiltration of interferon (IFN)-γ^+^ CD8 T cells, as depletion of this population abrogated this effect. Mice treated with the 11-strain mix also demonstrated lower frequency of colitis in response to anti-PD-1 therapy, suggesting that these bacteria could both enhance treatment efficacy and limit colon-specific side effects.

**Table 1:** Taxa associated with immunotherapy response

	Therapy	Response	Study
**PD-L1-associated taxa**			

*Bifidobacterium breve*, *B. longum*, *B. adolescentis*	10F.9G2	promoted anti-PD-L1 response; synergistically inhibited tumor growth	Sivan et al.^126^
*Akkermansia muciniphila*	nivolumab	abundance positively associated with treatment response in clinical and mouse fecal transplant models	Routy et al.^131^
*Ruminococcaceae Faecalibacterium*	anti-PD-1	abundance positively correlated with treatment response; diversity also correlated with treatment response	Gopalakrishnan et al.^130^

**CTLA4-associated taxa**			

*Bacteroides fragilis*, *B. thetaiotaomicron*, Burkholderiales	ipilimumab	T cell responses associated with bacterial abundance positively correlated with efficacy of CTLA4 blockade; anti-CTLA4 antibody therapy promoted *B. fragilis* abundance	Vétizou et al.^128^

Several clinical trials are currently underway to evaluate these hypotheses^135^^,^^136^ (ClinicalTrials.gov: NCT04264975 and NCT04130763), and their outcomes could significantly alter the prognosis and management of mCRC. Preliminary results are promising, with renewed response to anti-PD-1 therapy seen in previous immunotherapy-refractory metastatic melanoma.^136^ Of ten patients with anti-PD-1 treatment refractory melanoma, three were able to demonstrate either PR or CR following fecal transplant from anti-PD-1 responders. Taken together, these data demonstrate that the microbiome is a critical tool in modulating the efficacy of immune-based therapies and that they may soon be not just in the laboratory but also in the clinic.

### Conclusions

The advent of immunotherapy—in the active sense with drug intervention that stimulates the innate immune system to better recognize and counteract tumors through recognition of their antigens or cell surface markers—has changed the paradigm of treatment of solid tumors such as melanomas and non-small cell lung cancers and in the process of current investigation, challenging traditional notions that chemotherapy for CRC in general, and radiation for rectal cancers, needs to be the mainstay of treatment. Current progress most prominent with ICIs, and also CTLA-4 inhibitors and various forms of cellular therapy that are actively being investigated, is beginning to make an impact and will provide better knowledge for how these modalities may improve upon current strategies for intent-to-cure neoadjuvant therapies for these diseases. The current frontier includes many investigators aspiring to unlock and validate new markers beyond dMMR-MSI-H that will predict susceptibility to ICI and other immunotherapies. Understanding and differentiating biologic differences of these and similar populations in the context of molecular drivers, and their overall impact in different stages of carcinogenesis as well as in cases of CRC at different anatomic stages of progression, will be essential. Emerging knowledge of the effects of the microbiome, not just on CRC biology at all stages but also on modulating the efficacy of immune-based therapies, is another angle that will need attention in correlative studies to clinical trials as well as in design of the therapeutic approaches to care in all settings, including neoadjuvant approaches. Growing understanding in complex interactions between tumor cells and the immune system, analyzed in the context of the dynamic microbiome, all assessed using more sophisticated molecular and genetic techniques will merge our understanding and be a major basis for developing a better understanding of this process and will need to be taken into account for rational design of better and more strategically savvy therapeutic clinical trials.
